# Medicinal plants for the treatment and prevention of post-menopausal obesity: a review

**DOI:** 10.3389/fphar.2025.1564131

**Published:** 2025-05-21

**Authors:** Jing Liu, Reshmi Akter, Esrat Jahan Rupa, Hoang Van-An, Jinfeng Li, Deok Chun Yang, Dong Uk Yang, Muhammad Awais, Jong-Hoon Kim

**Affiliations:** ^1^ Jilin Ginseng Academy, Changchun University of Chinese Medicine, Changchun, China; ^2^ Graduate School of Biotechnology, College of Life Sciences, Kyung Hee University, Yongin‐si, Gyeonggido, Republic of Korea; ^3^ Mam Da Inside Joint Stock Company, Hanoi, Vietnam; ^4^ Shanghai Kangfu Enterprise Management Co., LTD., Shangai, China; ^5^ AIBIOME, Daejeon, Republic of Korea; ^6^ Department of Biotechnology, College of Fisheries Sciences, Pukyong National University, Busan, Republic of Korea

**Keywords:** estrogen, medicinal herbs, phytoestrogen, menopause, obesity

## Abstract

**Ethnopharmacological relevance:**

Phytoestrogen-rich plants have been used across various traditional medicine systems, such as Ayurveda, Traditional Chinese Medicine, and Indigenous herbal practices, to address menopausal symptoms including metabolic imbalances and weight gain. The historical use of these plants underscores their therapeutic potential in women’s health, providing a foundation for exploring their modern applications as safer alternatives to hormone replacement therapy (HRT) for post-menopausal obesity.

**Aim of the review:**

This review aims to systematically evaluate the anti-obesity effects of plant-derived phytoestrogens in managing post-menopausal obesity. It seeks to understand and summarize the mechanisms by which phytoestrogens act as estrogen alternatives, focusing on their cellular and molecular effects, and highlighting specific plants with promising therapeutic properties.

**Materials and methods:**

A comprehensive literature search was conducted, covering studies on phytoestrogenic plants used in traditional and contemporary practices for managing obesity. The review examines each plant’s taxonomic family, common name, bioactive compounds, and experimental evidence from cellular and animal models that illustrate potential anti-obesity mechanisms relevant to post-menopausal conditions.

**Results:**

The analysis reveals that phytoestrogens employ diverse mechanisms in mitigating obesity. Some bind directly to estrogen receptors, mimicking estrogenic effects and inducing cellular responses linked to metabolism. Others inhibit adipogenesis (fat cell formation) and lipogenesis (fat storage), while some enhance thermogenesis (heat production) and lipolysis (fat breakdown), effectively counteracting the metabolic shifts associated with menopause. Specific plants, such as soy (*Glycine max* (L.) Merr.), red clover (*Trifolium pratense* L.), and basil-clove (*Ocimum gratissimum* L.), demonstrate unique pathways for influencing fat metabolism, suggesting a multi-faceted approach to post-menopausal obesity.

**Conclusion:**

Plant-derived phytoestrogens have been proposed as a potential alternative to HRT for managing post-menopausal obesity. Drawing from both traditional ethnobotanical knowledge and emerging scientific evidence, these compounds may offer a naturally derived strategy that could carry fewer adverse effects. Nevertheless, current findings are preliminary, and more rigorous, large-scale clinical studies are necessary to better understand their efficacy, determine appropriate dosing, and assess possible interactions with conventional therapies.

## Graphical Abstract

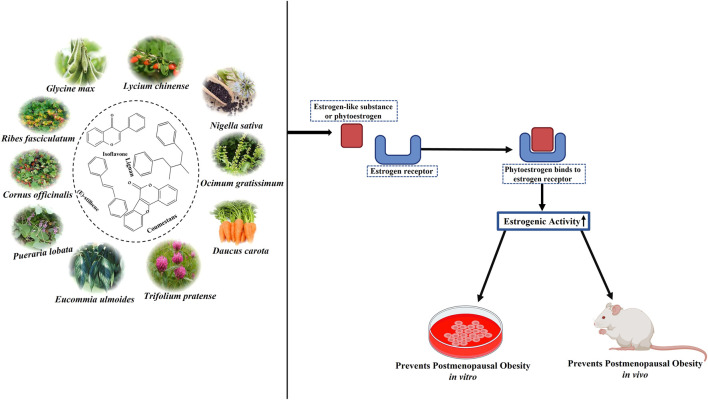

## Highlights

1. Energy imbalance, genetics, and lifestyle drive global obesity’s rise and impact.
2. Medicinal herbs boost ERα/ERβ expression, aiding post-menopausal estrogen activity.
3. Phytoestrogens mimic estrogen, aiding menopause and reducing various health risks.
4. Herbal extracts aid liver health and prevent obesity complications in menopause.
5. Browning of white adipose tissue boosts energy expenditure and combats obesity.

## 1 Introduction

Menopause, a natural biological stage in a woman’s life, marks the cessation of menstruation and the end of reproductive capacity. This phase, typically occurring between the ages of 40 and 58, is primarily characterized by a decline in estrogen levels and ovarian function depletion (Newson, 2018). In addition, menopause can be induced surgically through ovary removal or result from radiation or chemotherapy (Zhu et al., 2016). Post-menopause refers to the period when a woman has experienced 12 consecutive months without menstruation following her final menstrual cycle. This phase is often associated with various health concerns due to low estrogen levels, including cardiovascular disease, diabetes, non-alcoholic fatty liver disease, hypertension, osteoporosis, and notably, obesity or weight gain (Dalal and Agarwal, 2015; Nappi and Cucinella, 2020). Estrogen plays a crucial role in regulating fat distribution in the body by influencing insulin sensitivity in the liver, pancreas, and skeletal muscles, as well as the differentiation of white adipose tissue (WAT) and the induction of thermogenesis in brown adipose tissue (BAT) (López and Tena-Sempere, 2015). The estrogen deficiency during menopause leads to an imbalance between food intake and energy expenditure, resulting in increased energy storage and altered body fat distribution, particularly the accumulation of visceral fat (Butera, 2010). Post-menopausal women tend to have higher amounts of visceral body fat than their pre-menopausal counterparts. This excessive visceral fat is linked to insulin resistance and inflammation, contributing to metabolic disorders such as cardiovascular disease, type 2 diabetes mellitus, and non-alcoholic fatty liver disease (Ko and Jung, 2021). Furthermore, there is a positive correlation between obesity and an elevated incidence of invasive breast cancer among postmenopausal women (Qureshi et al., 2020). Therefore, managing and preventing post-menopausal obesity is of great importance for improving the quality of life and health in the post-menopausal population.

Hormone replacement therapy (HRT) has been a common choice for women to address declining estrogen levels since the 1970s (Akter et al., 2022). Although effective in alleviating menopausal symptoms, multiple clinical studies have highlighted its association with increased risks of breast cancer, stroke, and cardiovascular diseases, despite its cost-effectiveness (Hill et al., 2016). Moreover, various obesity medications have been approved by the US Food and Drug Administration (FDA) over the years. Unfortunately, some of these drugs were later withdrawn due to adverse effects. Currently, six drugs (Orlistat, Phentermine-topiramate, Naltrexone-bupropion, Liraglutide, Semaglutide, and Setmelanotide) are approved for long-term use (>12 weeks) in weight loss management (Müller et al., 2022). Most of these drugs primarily act on the central nervous system to suppress appetite and enhance satiety, while also exerting secondary effects on the gastrointestinal tract by delaying gastric emptying. All anti-obesity medications contribute to weight reduction and metabolic improvements, though their potency and effects vary depending on the specific drug (Chakhtoura et al., 2023). These medications, however, can cause side effects, including headache, dizziness, fatigue, nausea, constipation, dry mouth, diarrhea, vomiting, and dyspepsia. Consequently, there are no synthetic long-term, risk-free therapies available for managing post-menopausal obesity (Tak and Lee, 2020). In this context, the use of medicinal plants is increasingly gaining popularity. Medicinal plants are often more affordable, accessible, and consumed locally, either as raw materials or in simple medicinal preparations. Several studies have suggested that certain medicinal plants may possess potential anti-obesity effects of various medicinal plants. Notable examples include *Nigella sativa* L. (NS) (Naghsh et al., 2023), *Camellia sinensis* (L.) Kuntze (Laoung-On et al., 2024), *Dendropanax morbiferus* H.Lév. (Awais et al., 2023), *Panax ginseng* C.A.Mey. (Chen et al., 2025), among others. However, to our knowledge, there are no approved medicinal plants/drugs specifically designed for the treatment of obesity on the market.

Traditional, complementary, and integrative medicine is prevalent in nearly every country worldwide, serving as the primary healthcare system for many populations since ancestral times, with a growing demand for its services and an estimated 76% of the global population using some form of it each year (Hoenders et al., 2024). In western countries, approximately 40%–50% of women opt for complementary therapies, including plant-based treatments for menopause symptoms (Franco et al., 2016). This trend has garnered significant interest from the scientific community in exploring medicinal herbs as viable options for addressing menopause and its associated complications, including obesity (Balkrishna et al., 2024). This review aims to provide an updated perspective on the interplay between medicinal plants and post-menopausal obesity. It focuses on evaluating the efficacy of various therapeutic herbs rich in phytochemicals and phytoestrogens, emphasizing the underlying mechanisms in combating this condition.

## 2 Methodology

For a comprehensive understanding of the role of medicinal plants in managing post-menopausal obesity, a bibliographic search was conducted using Google Scholar, PubMed, ResearchGate, Scopus, and ScienceDirect with the keywords phytoestrogen and post-menopausal obesity. To obtain a holistic view of medicinal plants involved in estrogenic activity related to post-menopausal obesity, both recent and older papers published exclusively in English were accessed.

**FIGURE 1:**
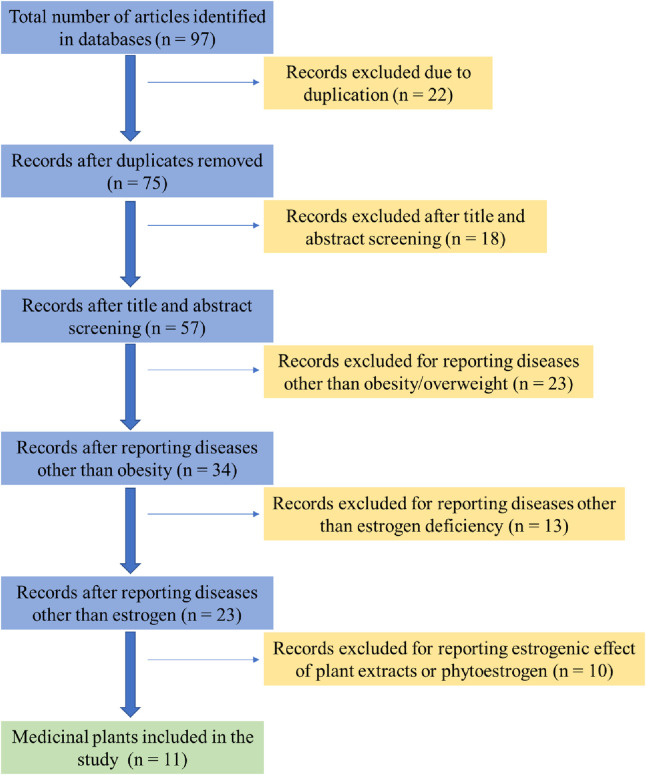
Flow chart summarizing exclusion and inclusion of medicinal plants as potential sources of phytoestrogen.

A preliminary screening of titles and abstracts was performed to eliminate studies with minimal or no relevance to the topic. The inclusion criteria focused on studies examining plants with synergistic effects on obesity and estrogen deficiency-related diseases (Figure 1). Highly relevant articles were thoroughly reviewed and summarized, with special attention given to the results and discussion sections. Additionally, key experimental studies (both *in vivo* and *in vitro*) and clinical investigations obtained from clinicaltrials.gov were compiled and are presented in both tabular and narrative formats.

## 3 Pathophysiology of post-menopausal obesity

**FIGURE 2:**
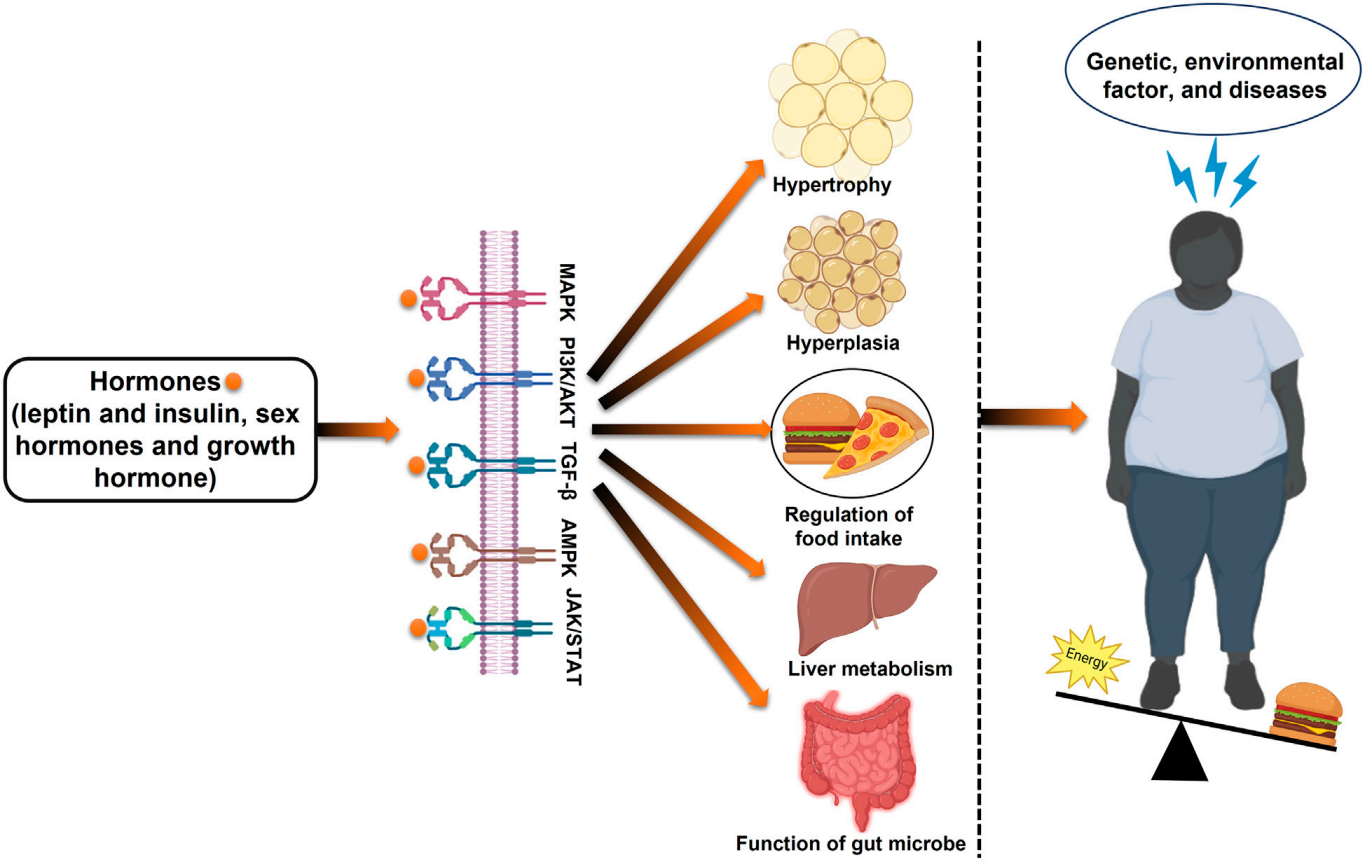
Schematic overview of the development of obesity: examining the interplay of metabolism, energy intake, genetics, and epigenetics.

Obesity is a complex metabolic disorder resulting from genetic, hormonal, environmental, and behavioral factors. It is primarily driven by an energy imbalance where caloric intake exceeds expenditure, leading to excessive fat accumulation (Clemente-Suárez et al., 2023). Beyond lifestyle and genetic predispositions, emerging evidence highlights the role of gut microbiota dysbiosis, epigenetic modifications, and hormonal dysregulation in the pathogenesis of obesity (Patra et al., 2023; Mazza et al., 2024). Among these factors, estrogen deficiency is particularly relevant in post-menopausal obesity (Figure 2).

**FIGURE 3:**
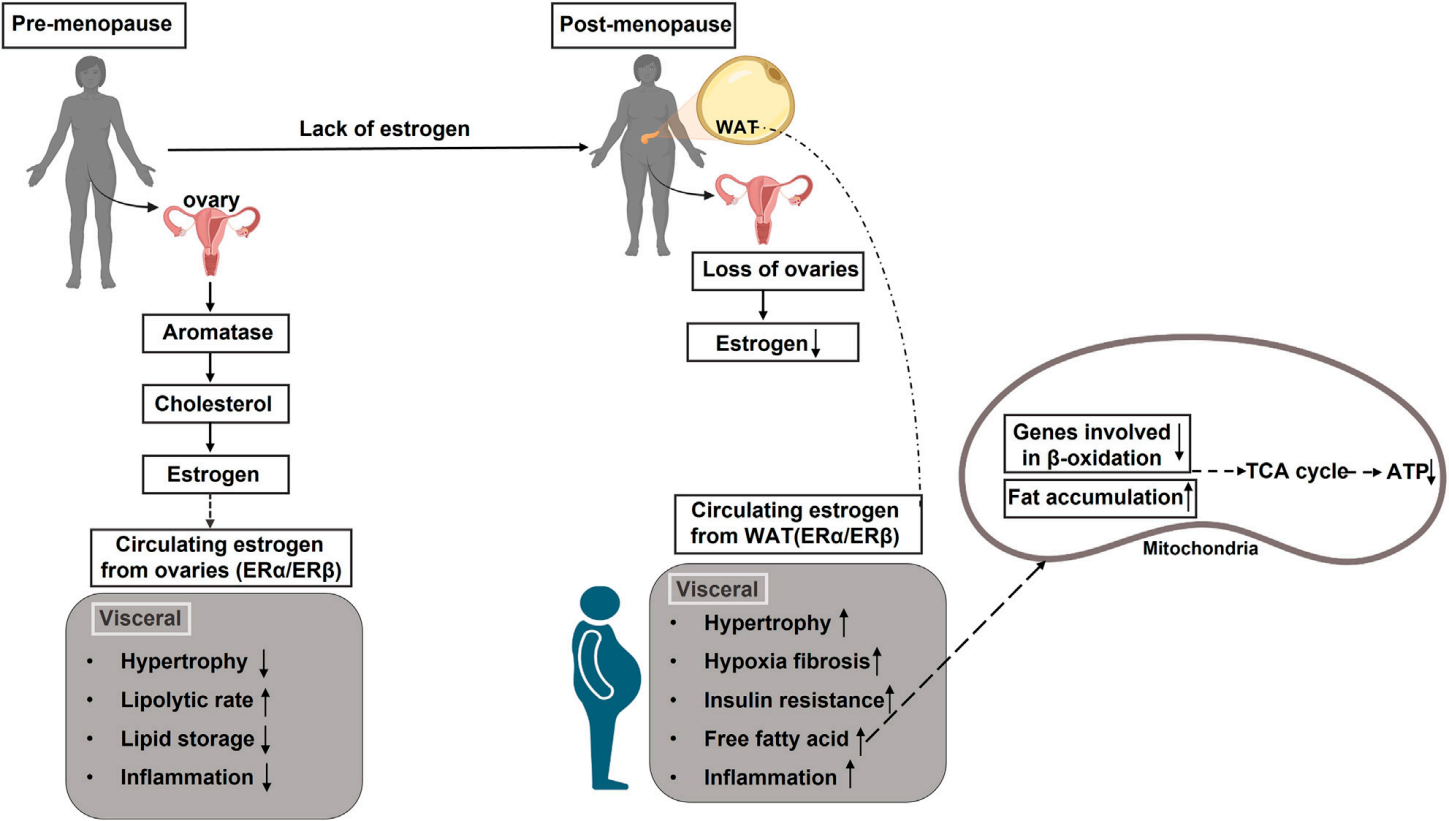
Impact of estrogen levels on adipose tissue distribution and metabolic health in pre-menopausal and post-menopausal females. In pre-menopausal women, ovarian-produced estrogen influences adipose tissue distribution by downregulating androgen receptors, promoting subcutaneous fat expansion while inhibiting visceral fat development. However, post-menopausal women experience a sharp decline in estrogen levels due to cessation of ovarian function. This reduction impairs estrogen receptor activation, leading to increased metabolic risks, including insulin resistance, dyslipidemia, and systemic inflammation. These changes, combined with the downregulation of beta-oxidation genes, result in an excess of free fatty acids that cannot be efficiently utilized for energy production.

Estrogen plays a central role in adipose tissue metabolism, influencing fat distribution, energy expenditure, and inflammatory pathways. In pre-menopausal women, estrogens regulate lipid metabolism through estrogen receptors (ESR1 and ESR2), promoting beta-oxidation and limiting adipogenesis (Gregorio et al., 2021). However, the decline in estrogen levels after menopause shifts fat deposition from a gynoid (hip-thigh) pattern to an abdominal (visceral) pattern, increasing metabolic risks (Maheswari and PE, 2022). This transition is associated with reduced mitochondrial activity, decreased BAT thermogenesis, and increased WAT storage, further exacerbating related metabolic complications (Pu et al., 2021; Malik et al., 2024) (Figure 3).

**FIGURE 4:**
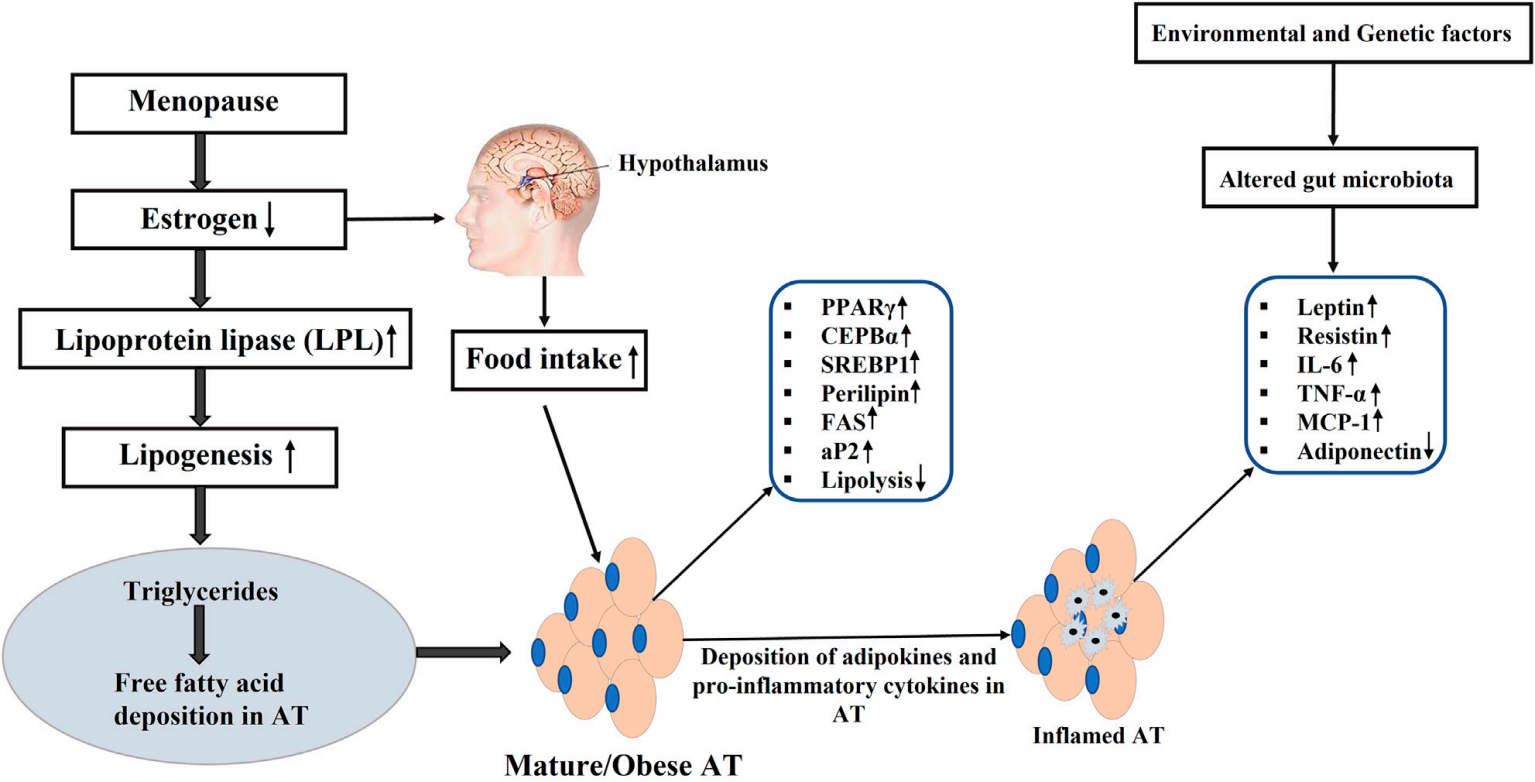
Estrogen deficiency and origin of obesity. PPARγ: Peroxisome proliferator-activated receptor γ; C/EBPα: CCAAT-enhancer-binding protein α; C/EBPβ: CCAAT-enhancer-binding protein β; SREBP-1c: Regulatory element binding protein-1c; *Plin1*: Perilipin 1; FAS: Fatty-acid synthase; aP2: Adipocyte protein 2; IL-6: interleukin 6; MCP-1: The monocyte chemoattractant protein-1; TNF-α: tumor necrosis factor α; (↑): upregulation; (↓): downregulation.

Additionally, estrogen deficiency disrupts appetite regulation via hypothalamic signaling, contributing to altered food intake and energy homeostasis (González-García et al., 2023). It also impacts glucose metabolism by influencing key enzymes such as GLUT3 and GLUT4, thereby increasing insulin resistance and metabolic dysfunction (Chang et al., 2023). The decline in estrogen further leads to elevated pro-inflammatory adipocytokines (TNF-α, IL-6), increased low-density lipoprotein (LDL), and heightened triglyceride levels, all of which elevate the risk of cardiovascular disease and metabolic syndrome (MetS) (Abildgaard et al., 2021; Alves et al., 2025) (Figure 4).

Given the metabolic challenges posed by menopause, interventions targeting estrogenic pathways have gained interest. While HRT has been explored, concerns regarding its long-term safety have shifted attention toward medicinal plants with estrogenic and metabolic regulatory properties as potential therapeutic options for managing post-menopausal obesity.

## 4 Medicinal plants as a potential source of phytoestrogens

**FIGURE 5:**
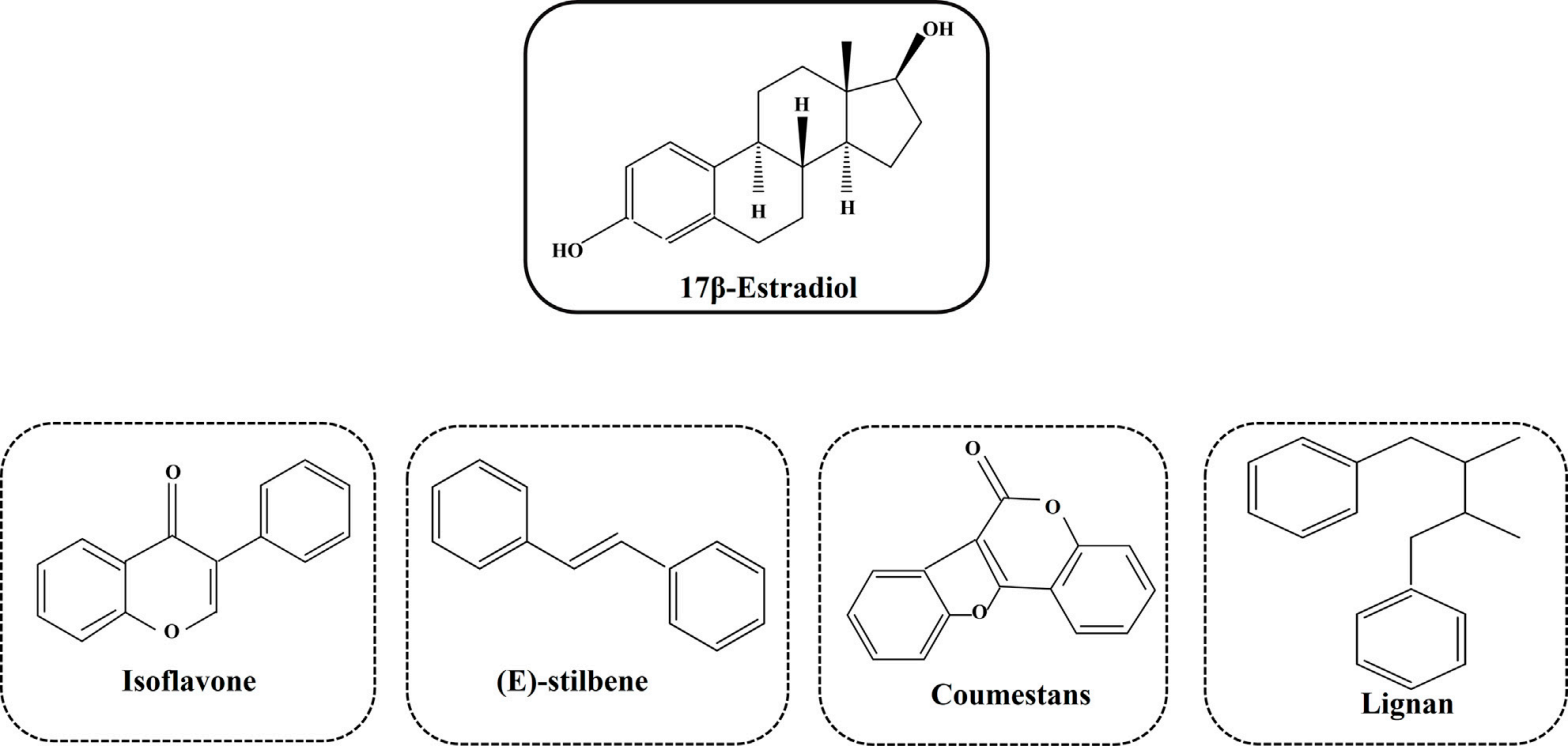
Different types of phytoestrogens and their structural similarities with synthetic estrogen.

Phytoestrogens are a diverse group of non-steroidal compounds of plant origin or biologically derived from plant precursors, are structurally similar to the primary female sex hormone, E_2_ (Yokosuka et al., 2021). Four phenolic compounds identified as phytoestrogens are isoflavones, stilbenes, coumestans, and lignans (Figure 5). They are found in a wide array of plants, primarily categorized into these four classes (Desmawati and Sulastri, 2019). Major sources of isoflavones include legumes such as *Glycine max* (L.) Merr. (soy) and its products, *Trifolium pratense* L. (TP, red clover), and NS (black cumin), while fiber-rich foods such as unrefined grains, cereal brans, and beans are abundant in lignans (Bedell et al., 2014). The most studied isoflavones, including genistein, daidzein, glycitein, formononetin, and biochanin A, are prevalent in soybeans. Although a diet low in phytoestrogens does not lead to any deficiency syndrome, given their structural resemblance to synthetic estrogen and their ability to bind to estrogen receptors, they hold therapeutic significance (Rietjens et al., 2017).

Phytoestrogens act as mild estrogen agonists that target certain receptors and cell types via ERα, ERβ, and other signaling pathways. Thus, with estrogen-like effects, phytoestrogens increase estrogen levels in the body and provide post-menopausal women with a practical alternative to synthetic HRT, which has certain side effects. Plant-based estrogen can reduce the risk of clotting and alleviate menopausal symptoms like hot flashes and osteoporosis, and many women have begun to use phytoestrogen supplements (Franco et al., 2016). Furthermore, exposure to phytoestrogens can reduce the risks of cardiovascular disease, obesity, MetS, type 2 diabetes mellitus, brain function disorders, breast cancer, and other malignancies, including prostate and colorectal cancers. However, the potential hormone-related side effects of phytoestrogen treatment, such as endometrial hyperplasia, endometrial cancer, and breast cancer, remain uncertain. Consequently, the safety and tolerance of long-term supplementation with phytoestrogens are under scrutiny. Additionally, other phytochemicals, such as triterpenoids, phenols, flavonoids, lignans, sterols, terpenoids, iridoids, polysaccharides, amino acids, unsaturated FAs, carbohydrates, and carotene, were found in *Lycium chinense* Mill. (LC), *Ocimum gratissimum* L. (OG), *Eucommia ulmoides* Oliv. (EU), *Pueraria lobata* (Willd.) Ohwi (PL), *Rehmannia glutinosa* (Gaertn.) DC. (RG), *Cornus officinalis* Siebold & Zucc. (CO), and *Daucus carota* L. (DC). These phytochemicals render the above plants pharmacologically important for treating various severe illnesses, including cancer. Table 1 lists all the medicinal plants reviewed in this article, along with their common names, families, and chemical components.

**TABLE 1:** Family, common name, distribution and phytochemical constituents of the studied medicinal plants.

Plant	Family	Common name	Distribution	Phytochemicals/phytoestrogen	Ref.
*Lycium chinense* Mill	Solanaceae	Chinese Boxthorn, Chinese Desert-thorn Chinese Matrimony Vine, Goji Berry, Chinese Wolfberry	China, Korea, and Japan	Polysaccharides: arabinose, galactose, glucose, rhamnose; Alkaloids: 3-hydroxy-4-ethyl ketone pyridine, indolyl-3-carbaldehyde; Glycopeptide: galacturonic acid peptides: lyciumins A-D (1–4) Tocopherols: α-Tocophero and Flavonoids: Quercetin	Lei et al. (2022)
*Pueraria lobata* (Willd.) Ohwi	Leguminosae	Kudzu, Ohwi, Galgeun	Asia, North America,and South America	Isoflavonoids: puerarin, daidzin, daidzein, and genistein	Prasain et al. (2003), Wang et al. (2020)
*Rehmannia glutinosa* (Gaertn.) DC.	Plantaginacea	Chinese foxglove, Dihuang, Rehmannia, Sheng Di Huang	China provinces like Shandong, Shanxi, Hebei, Henan, Shan’xi, Liaoning, Inner, Jiangsu, Zhejiang, Sichuan, Mongolia, Hubei, and Huna	Flavonoids: daidzein and genistein; Phenol: phenolic acids; Glycoside: jioglutosides A and B; Ionones: frehmaglutin F, frehmaglutin G Microelements: iron, zinc, manganese; Iridoids: 8-epiloganic acid and ajugol; Monoterpenes: jioglutins A, B, C, jioglutolide and jiofuran	Zhang et al. (2008)
*Cornus officinalis* Siebold & Zucc	Cornaceae	Shan-zhu-yu, Japanese cornel, dogwood	China, Korea, and Japan	Terpenoids: oleanolic acid; Flavonoids: Kaempferol and Quercetin; Tannins: ellagitannins, gallotannins; Phenylpropanoids: cornuphenylpropanoid A; Sterols: β-sitosterol Fatty acid: Lauric acid, Palmitic acid; furans, and mineral substances	Gao et al. (2021)
*Ribes fasciculatum* Siebold & Zucc	Grossulariaceae	Honshu, Shikok, Kyushu	China, Japan, and Korea	Polyphenolic compounds including catechin, gallocatechin, quercitrin	Dat et al. (2005)
*Glycine max* (L.) Merr	Fabaceae	Soybean, soya	China, Manchuria, Korea, Japan and other parts of Asia	Isoflavones: Daidzin, glycitin, genistein, daidzein; Protein: globulin, Oil content: fatty acid including palmitic acid and stearic acid; Carbohydrate: sucrose, maltose, glucose, fructose and 3 cylitols; Saponins: soyasaponin group A and B and non-digestible oligosaccharides	Xu et al. (2020)
*Ocimum gratissimum* L	Lamiaceae	Basil, basil-clove, or alfavaca	Tropical Africa, India and South East Asia, China, South America, the Caribbean, Australia, New Zealand and on many islands in the Indian and the Pacific region	Tannins: phlobatannin; Triterpinoids: pomolic, tormentic, oleanolic acid; Essential oil: eugenol, ethyl cinnamate, methyl eugenol, farnesene, bisabolene; Carbohydrate: uronic acid, pentoses; Phenol: thymol; and lipids	Ugbogu et al. (2021)
*Daucus carota* L	Apiaceae	Wild carrot, bird’s nest, bishop’s lace, and Queen Anne’s lace	Atlantic coast of Britain, Ireland through Europe and the Mediterranean to Central Asia	Flavonoids: apigenin, chrysin, luteolin, kaempferol, quercetin; Coumarins: osthole, bergapten, zosimin, and β-sitosterol; Anthocyanes: cyanidin, delphinidin, malvidin and pelargonidin; Terpenoids: β-caryophyllene, β-farnesene, β-bisabolene and caryophyllene oxide; Carbohydrate: fructose, glucose, mannitol and Vitamin: β carotene (vitamin A)	Hammami et al. (2019)
*Eucomma ulmoides* Oliv	Eucommiaceae	Oliver, Duzhong, hardy rubber tree	China, Japan, Korea, France, Russia, Canada, Germany, and India	Iridoids: asperuloside, geniposidic acid, aucubin; phenolic compound: Chlorogenic acid, flavonoids: eucommiaflavone, licoflavone; Sterols: β-sitosterol; Terpenoids: betulic acid, oleanolic acid; Polysaccharides: uronic acid; Unsaturated fatty acids: α-linolenic acid; and mineral elements	Xing et al. (2019)
*Trifolium pratense* L	Fabaceae	Red clover	Europe, Western Asia, and northwest Africa, North and South America	Isoflavones such as daidzein, genistein, biochanin A, and formononetin	Vlaisavljevic et al. (2014)
*Nigella sativa* L	Ranunculaceae	Black seed or black cumin	Middle East, Eastern Europe, and Western Asia	Quinones: Thymoquinone, 2-isopropyl-5-methyl-1,4-benzoquinone; Proteins: amino acids; Fixed oils: linoleic, oleic and palmitic acids and carbohydrates	Ijaz et al. (2017)

## 5 Experimental studies

Experimental studies, both *in vivo* and *in vitro*, have been conducted to explore the efficacy of various medicinal plants and their bioactive compounds in mitigating menopause and related post-menopausal disorders, such as obesity. The following discussion emphasizes the underlying mechanisms and actions of these plants in combating post-menopausal obesity, offering a deeper understanding of their roles and effects in this context.

### 5.1 Effect of medicinal plants on estrogen receptors

ERs (ERα and ERβ) are members of the nuclear receptor superfamily of ligand-regulated transcription factors, and they function as signal transducers and transcription factors, regulating the expression of the target genes (Yang et al., 2019). Following interaction with a ligand, these receptors can relocate from the cytoplasm to the nucleus and bind to transcription-regulatory regions of DNA or short RNAs, thereby regulating the expression of specific genes (Sirotkin and Harrath, 2014). Due to their structural resemblance to estrogen, phytoestrogens can interact with ERs, stimulating or inhibiting estrogenic responses. This interaction is particularly relevant for obese post-menopausal women using phytoestrogens. Soy isoflavones, natural selective estrogen receptor modulators, have been shown to reduce lipid accumulation and adipose tissue distribution (Wei et al., 2012). Reproductive hormones are typically produced in the ovaries and are essential for maintaining female sex characteristics as well as fertility, pregnancy, and the menstrual cycle. Therefore, the OVX mice are commonly used to assess the effects of all medicinal plants and their bioactive components on weight gain and metabolic profiles. This procedure induces biochemical changes in menopause, and it helps investigate the long-term effects of reduced estrogen levels (Rodríguez-Landa, 2022). The experimental animals are divided into three groups: sham-operated (placebo surgery), which is a sham surgical intervention omitting the step considered therapeutically necessary, OVX, and OVX animals treated with estrogen-like substances.

The uterus, comprising various cell types, including smooth muscle, stroma, glandular, and luminal epithelia, plays a crucial role in regulating circulating estrogen and progesterone levels to control morphological changes. Uterine ERα is particularly important in mediating the effects of estrogen (Cheon et al., 2009). In OVX mice, the surgical removal of ovaries leads to thinning of the uterus due to reduced estrogen production. The disrupted translation of uterine estrogen receptors results in impaired regulation of lipid metabolism. Nevertheless, the weight of the uterus and the expression of ERα/ERβ in the uterus was enhanced in post-menopausal obese mice after LC extract treatment. Furthermore, granulosa cells in the follicles of mammalian ovaries continue to release more estrogen as the follicles develop (Kono et al., 2014). The mouse *ERα* mRNA expression in the murine uterus and estradiol production in COV434 granulosa cells were both enhanced by the CO and *Ribes fasciculatum* Siebold & Zucc. (RF) combination (CO + RF) extract. Hence, the administration of medicinal plants may promote estrogen-like activity and prevent ovariectomy-induced uterine atrophy by upregulating mouse *ERα* (ESR1) and *ERβ* (ESR2) (Table 2).

**TABLE 2:** *In vivo* and *in vitro* studies for different medicinal plants and their phytoestrogen in post-menopausal obesity.

Plant	Extract/compound	Model	Mechanisms	Ref.
*Lycium chinense* Mill	Aqueous extract	C57BL/6 mice, 3T3-L1 preadipocytes	Body weight↓, fasting blood glucose levels↓, serum lipid↓, visceral fat weights↓, adipocyte size↓, lipid droplets in hepatocytes (hepatic steatosis), uterus weight↑, expression of ER-α/ER-β in the uterine↑, PPARγ↓, GLUT4↑, plin↓, ER-α↑, ER-β↑	Kim et al. (2017)
Combination of *Pueraria lobata* (Willd.) Ohwi and *Rehmannia glutinosa* (Gaertn.) DC. (HT051)	Aqueous extract	Ovariectomized (OVX) rats	body fat↓, hyperlipidemia↓, hyperglycemia↓, AST↓, ALT↓, SREBP1-c↓, FAS↓, PPAR*α*↑, CTP-1↑, PPAR*γ*↓, aP2↓, IL-6↓, MCP-1↓	Lee et al. (2017)
*Cornus officinalis* Siebold & Zucc. and *Ribes fasciculatum* Siebold & Zucc	Provided by Plant Extract Bank in the Korea Research Institute of Bioscience and Biotechnology	Ovariectomized (OVX) rats, 3T3-L1 preadipocytes	body weight↓, total fat↓, abdominal visceral fat↓, serum leptin, insulin levels↓, of ER-α in the uterine↑, *Plin1*↓, Adipoq↓	Park et al. (2020)
*Glycine max* (L.) Merr	Prethanol extract	Ovariectomized (OVX) mice, 3T3-L1 preadipocytes	weight gain↓, adipocyte area↓, serum metabolic profiles↓, hypertrophy↓, hyperplasia↓, weight gain↓, adipocyte area↓, serum metabolic profiles↓, C/EBP-α↓, PPAR-γ↓, C/EBP-β↓, C/EBP-α↓, SREBP1-c↓, caveolin1↓, Plin↓, UCP-1↑, CIDEA↑, PRDM16↑, CPT-1↑, PGC-1α↑, CD137↑, Tbx-1↑ ACOX1↑, ATGL↑, ACSL1↑	Xie et al. (2018), Kim et al. (2019)
*Ocimum gratissimum* L	aqueous extract	female Sprague–Dawley rats	body weight↓, adipocyte size↓, bone mass↑, body composition↑	Chao et al. (2017)
*Daucus carota* L	aqueous extract	Female Sprague–Dawley rats	Fat mass↓, weight gain↓, hepatic TG levels↓, CPT-1↑, PPAR-α↑, FAS↓, SREBP-1c↓	Park et al. (2015)
*Eucommia ulmoides* Oliv	aqueous extract	Ovariectomy (OVX) rats	body weight↓, BMI↓, BMD↑	Zhang et al. (2012)

PPARγ: Peroxisome proliferator-activated receptor γ; C/EBPα: CCAAT-enhancer-binding protein α; C/EBPβ: CCAAT-enhancer-binding protein β; SREBP-1c: Regulatory element binding protein-1c; Glut4: Glucose transporter type 4; *Plin1*: Perilipin 1; FAS: Fatty-acid synthase; aP2: Adipocyte protein 2; CIDEA: Cell Death Inducing DFFA, Like Effector A, PGC-1α: Peroxisome proliferator-activated receptor α; PRDM16: PR, domain containing 16; UCP-1: Uncoupling protein; CD137: tumor necrosis factor receptor superfamily member 9; Tbx-1: T-Box Transcription Factor 1; ACOX1: Peroxisomal acyl-coenzyme A oxidase 1; CPT-1a: Carnitine palmitoyltransferase I; ATGL: adipose triglyceride lipase; ACSL: Acyl-CoA, Synthetase Long Chain Family Member 1; ER-α: Estrogen receptor α; ER-β: Estrogen receptor β; IL-6: interleukin 6; MCP-1: The monocyte chemoattractant protein-1; AST: aspartate aminotransferase; ALT: Alanine aminotransferase; BMI: Body mass index; BMD: Bone mass index; TG: Triglyceride (↑): upregulation; (↓): Downregulation.

### 5.2 Effect of medicinal herbs on obesity-related parameters

**FIGURE 6:**
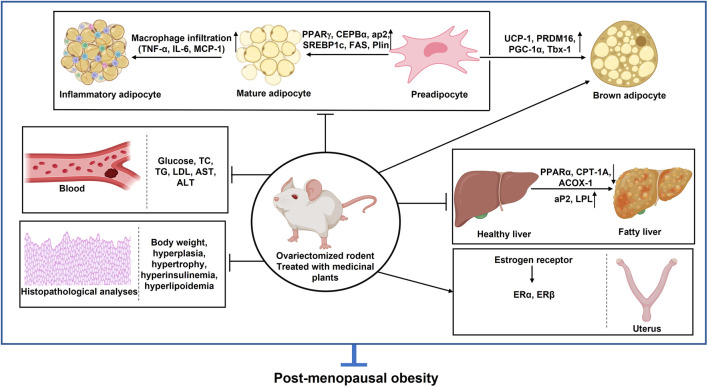
*In vivo* studies for different plants and their phytoestrogen in post-menopausal obesity. PPARγ: Peroxisome proliferator-activated receptor γ; C/EBPα: CCAAT-enhancer-binding protein α; C/EBPβ: CCAAT-enhancer-binding protein β; SREBP-1c: Regulatory element binding protein-1c; Glut4: Glucose transporter type 4; *Plin1*: Perilipin 1; FAS: Fatty-acid synthase; aP2: Adipocyte protein 2; PGC-1α: Peroxisome proliferator-activated receptor α; PRDM16: PR domain containing 16; UCP-1: Uncoupling protein; CD137: tumor necrosis factor receptor superfamily member 9; Tbx-1: T-Box Transcription Factor 1; ACOX1: Peroxisomal acyl-coenzyme A oxidase 1; CPT-1a: Carnitine palmitoyltransferase 1a; ER-α: Estrogen receptor α; ER-β: Estrogen receptor β; IL-6: interleukin 6; MCP-1: The monocyte chemoattractant protein-1; AST: aspartate aminotransferase; ALT: Alanine aminotransferase; BMI: Body mass index; BMD: Bone mass index; TG: Triglyceride (↑): upregulation; (↓): Downregulation.

The primary contributor to being overweight and obesity is an ongoing imbalance between energy intake and energy expenditure, leading to expanded adipose tissue that can store an excessive amount of energy. Adipose tissue growth occurs through two mechanisms: an increase in cell number (hyperplasia) and an increase in cell size (hypertrophy) (Jo et al., 2009). Overweight and obesity are major risk factors for several chronic diseases, such as diabetes, hypertension, and cancer. Body mass index (BMI) is a common measure for classifying overweight and obesity in adults, while elevated waist circumference is used to identify abdominal obesity, an independent health risk predictor. Post-menopausal women often experience significant visceral fat gain and are overweight, which is closely linked to estrogen deficiency (Sirotkin and Harrath, 2014). This increase in fat heightens the risk of MetS, leading to hypertension, insulin resistance, and dyslipidemia—an imbalance of lipids such as cholesterol, LDL-C, TGs, and HDL (Kaaja, 2008). The deficiency of estrogen in post-menopausal women may be a significant contributing factor to obesity. Thus, controlling obesity-related parameters during the post-menopausal stage is considered an effective approach to prevent post-menopausal obesity. In this review, we discuss the properties of several medicinal plants, phytoestrogen, and plant-based chemical extracts against estrogen-deficient obesity. The majority of studies show that anti-obesity agents lead to weight loss or changes in body fat in both OVX animals and humans. The plants induced significant weight loss and improvements in lipid profiles in the animals, with increased HDL-C levels and reduced TG, total cholesterol (TC), and LDL-C levels. Aqueous extracts from plants such as LC, a combination of PL and RG (HT051), OG, and DC have been shown to reduce weight gain, BMI, adipocyte area, adipocyte size, and LDL while increasing HDL in animals treated with the aqueous plant extracts (*in vivo*) (Figure 6).

For cellular studies, the 3T3-L1 preadipocyte cell line was used to determine lipid accumulation and gene and protein expression profiles. This cell line has been widely utilized in adipogenesis and adipocyte biochemistry research due to its capacity to differentiate fibroblasts from adipocytes (Zebisch et al., 2012). Treatments with the mentioned medicinal plants reduce hypertrophy and hyperplasia *in vitro*. Moreover, the soyasaponin Ab (SA) fraction, significantly elevated in germinated soy germ extract (GSGE), has been shown to prevent the formation of lipid droplets (LD) and lipid accumulation in differentiated 3T3-L1 cells. Soyasaponins and soyasapogenol are known to affect weight gain, decrease adipose tissue, and improve blood lipid and glucose profiles (triglyceride, cholesterol, and glucose).

### 5.3 Medicinal plants suppress adipogenesis and lipogenesis

Numerous studies have focused on the inhibitory effects of medicinal plants on adipogenic differentiation, the process by which preadipocytes transform into mature adipocytes, and on lipogenesis, the synthesis of fat in these cells. Key early-phase adipogenesis transcription factors, C/EBPβ and C/EBPδ, induce the expressions of PPARγ and C/EBPα (Bjune et al., 2022). These, in turn, promote the expression of genes involved in fat metabolism, including sterol-regulatory-element-binding protein 1c (SREBP-1c), the glucose transporter GLUT4 (also known as SLC2A4), fatty-acid-binding protein (FABP4, also known as aP2), LPL, adiponectin, and leptin (Lowe et al., 2011). Therefore, targeting adipogenesis presents a potential strategy for combating obesity.

Sex steroid hormones significantly influence the metabolism, development, and distribution of adipose tissue (Jang et al., 2024). E_2_ has been identified as a major regulator in the metabolism of female adipose tissue. Through the stimulation of mTOR signaling and PPAR inhibition, estrogen has been demonstrated to decrease adipogenesis. Medicinal plant extracts, such as those from DC and a combination of PL and RG (HT051), have notably suppressed adipogenic transcription markers, including PPARγ, aP2, SREPB-1c, and fatty acid synthase (FAS) in OVX rats. Lipolysis is the enzymatic process of hydrolyzing TGs into FAs and glycerol. It involves lipases and is regulated by perilipin (Plin), which limits lipase access to TGs (Edwards and Mohiuddin, 2020). Both *in vitro* and *in vivo* studies have shown that extracts from LC and CO + RF suppress PPARγ and Plin (Table 2). Estrogen regulates glucose/energy metabolism by influencing the expression of enzymes (Glut3 and Glut4) involved in glucose metabolism (Chen et al., 2018). In estrogen-deficient conditions, GLUT4’s glucose transport efficiency is reduced, but LC treatment has been found to elevate GLUT4 expression, facilitating glucose removal from adipose tissues. Modulators of adipocyte lipid metabolism like perilipin 1 (*Plin1*) and *Adipoq* control adipogenic differentiation (De Jager et al., 2013). The expression of the new *Adipoq* gene is noticeably elevated in differentiated 3T3-L1 cells. However, CO + RF treatment significantly downregulated the mRNA expression levels of *Plin1* and *Adipoq* in differentiated 3T3-L1 cells.

**FIGURE 7:**
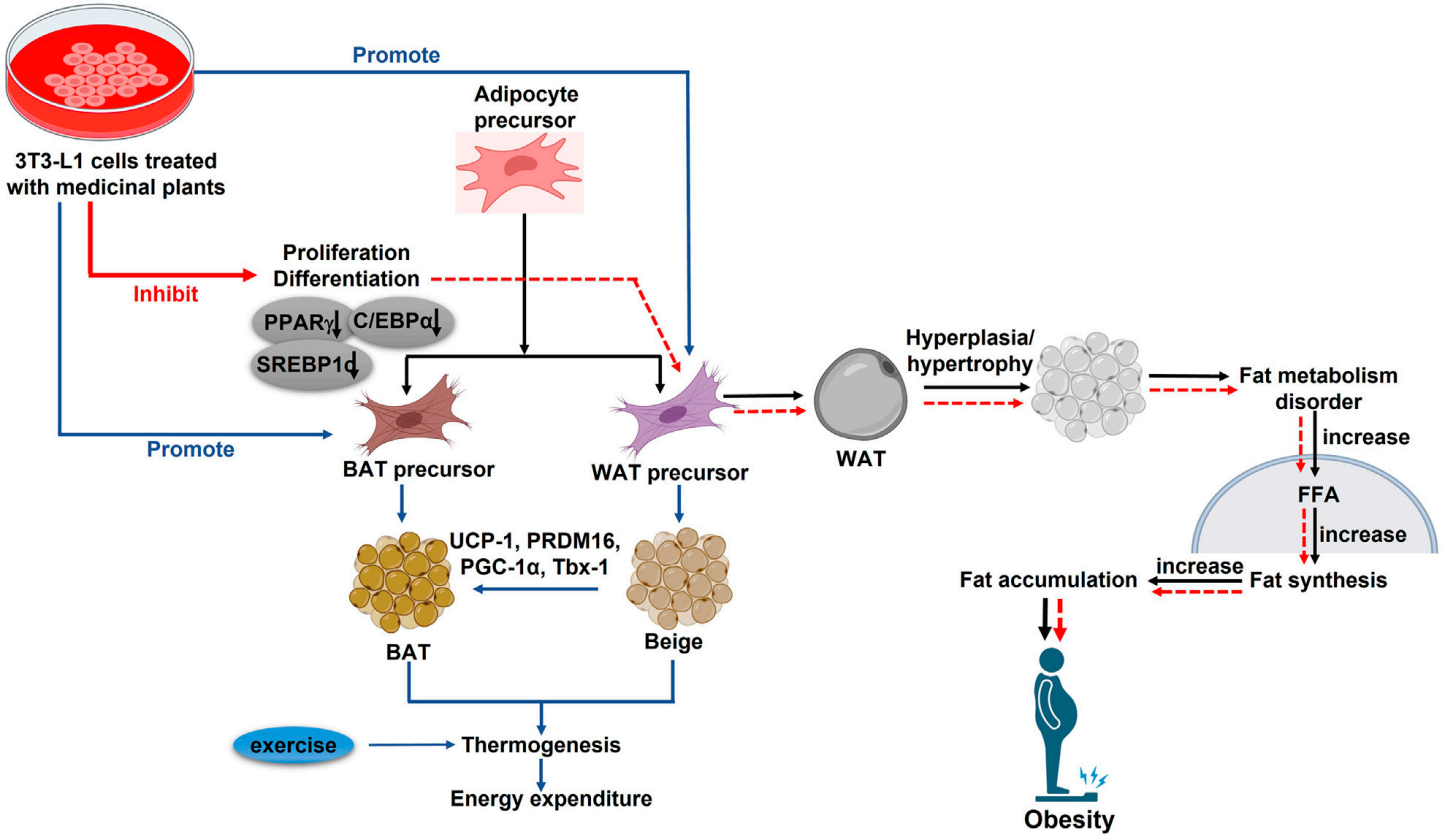
*In vitro* studies for different plants and their phytoestrogen in post-menopausal obesity. PPARγ: Peroxisome proliferator-activated receptor γ; C/EBPα: CCAAT-enhancer-binding protein α; SREBP-1c: Regulatory element binding protein-1c; PGC-1α: Peroxisome proliferator-activated receptor α; PRDM16: PR domain containing 16; UCP-1: Uncoupling protein; Tbx-1: T-box protein 1; WAT: White adipose tissue; BAT: Brown adipose tissue (↑): upregulation; (↓): Downregulation.

Lipogenesis refers to the production of FAs and TGs from substrates like glucose. TGs will be produced by lipogenesis and stored in adipose tissue as a source of energy. These TGs can later be broken down by beta-oxidation to produce adenosine triphosphate (ATP) (Musselman et al., 2013). Gene and protein expression analyses revealed that lipogenic genes and proteins, such as Stearoyl-CoA Desaturase 1 (SCD1), acetyl CoA carboxylase (ACC), and FAS were significantly lower in GSGE and SA groups than in the non-treated group, leading to reduced lipogenesis *in vivo*. Furthermore, triacylglycerols (TAGs) are hydrolyzed to glycerol and free FAs (FFAs) during lipolysis. FFAs are transported and absorbed by different tissues after being released into the circulation to be used for oxidation and ensuing ATP synthesis. While some FFAs are re-esterified into intracellular TAG (Ahmadian et al., 2010). SA treatment increased the expression of adipose triglyceride lipase (ATGL), a lipolytic protein. To a great extent, the authors determined the effect of SA/GSGE on LD formation as well as TG metabolism in an animal model. SA/GSGE administration also reduced the expression of LD formation- and fusion-related genes, such as *caveolin 1*, *caveolin 2*, *fat-specific protein 27* (*FSP27*), *Plin1*, and *CGI-58*, along with caveolin 1 and Plin protein levels. Acyl-CoA Synthetase Long Chain Family Member 1 (ACSL1), a TG synthesis gene, was also downregulated by SA/GSGE treatment. Enhanced endocannabinoid system (ECS) activity is associated with higher food intake and weight gain in animals (Nesto and Mackie, 2008). The study examined whether SA treatment alters the ECS, and the results revealed that ECS-related protein expression was notably altered by SA. Additionally, cannabinoids can activate the CB1 receptor, and N-acyl phosphatidylethanolamine-specific phospholipase D [NAPE-PLD], a CB1-synthesis enzyme, was downregulated by SA treatment in OVX mice. Conversely, CB2, diacylglycerol lipase (DAGL-α), monoacylglycerol lipase (MAGL), and other CB2-related genes and proteins which contribute to anti-obesity effects by reducing food intake (Rossi et al., 2018), were elevated by SA/GSGE treatment (Figures 6, 7). These findings disclose that the anti-obesity effect in an estrogen-deficient state is accompanied by ECS modulation.

In summary, these results suggest that the medicinal plants discussed exhibit similar gene regulatory effects, implying that plant extracts rich in phytoestrogens could potentially exert anti-obesity effects in estrogen-deficient women.

### 5.4 Medicinal plants induce browning of WAT

Mammals possess two distinct types of adipose tissues, known as WAT and BAT, each playing distinct roles in regulating energy balance and homeostasis (Ikeda and Yamada, 2020). WAT primarily stores TGs and produces adipokines like leptin and adiponectin, while BAT, characterized by numerous small LD, releases chemical energy as heat through the action of UCP-1 in the mitochondria (Bargut et al., 2017). Interestingly, WAT can transform into a brown-like state, abundant in UCP-1 and BAT-specific markers, through a process known as “browning.” This transformation can be triggered by stimuli such as cold exposure, exercise, and adrenergic β3-receptor activation (Véniant et al., 2015). Targeting WAT browning may be a viable strategy to mitigate obesity, particularly in estrogen-deficient women. Estrogen is known to regulate thermogenesis in BAT and insulin sensitivity in organs like the liver, pancreas, and WAT (Park et al., 2018). Key markers of brown and beige adipocytes, such as UCP-1, PR domain containing 16 (PRDM16), peroxisome proliferator-activated receptor-gamma coactivator (PGC-1α), and T-box transcription factor (Tbx1), are elevated during WAT browning, along with mitochondrial biogenesis. PGC-1α, initially identified as a PPAR coactivator, plays a pivotal role in controlling the UCP-1 promoter in adipocytes and mitochondrial biogenesis, while PRDM16 is crucial in determining the density and function of brown fat cells. Isoflavones, a group of phytoestrogens most abundant in soy, and soyasaponin groups A and B are key compounds found in soybeans. SA treatment has been observed to increase the expression of thermoregulatory proteins, including UCP-1, PRDM16, and PGC-1α *in vitro*. This finding suggests that SA treatment promotes the browning of WAT, contributing to an anti-obesity effect in the absence of estrogen by increasing energy expenditure.

### 5.5 Medicinal plants combat adipose tissue inflammation

The complications arising from obesity are often exacerbated by inflammation in adipose tissues. This inflammatory state is characterized by immune cell infiltration, increased levels of pro-inflammatory cytokines, insulin resistance in adipocytes, mitochondrial dysfunction, and endoplasmic reticulum stress (Shen et al., 2019). Additionally, it triggers the activation of NF-κB signaling cascades. Local adipose tissue hypoxia induces the release of pro-inflammatory cytokines, such as chemokine MCP-1, TNF-α, and IL-6, which exacerbate metabolic disorders, including osteoporosis, liver diseases, atherosclerosis, and cancer (Akter et al., 2022). The overexpression of these cytokines can lead to systemic insulin resistance by impairing the function of IRS-1 and AKT, which are important regulators of glucose absorption, lipogenesis, and glycogen synthesis. The connection between inflammation and insulin resistance is a key aspect of obesity (Alemán et al., 2017). Estrogen acts as an anti-inflammatory agent, and its deficiency can amplify the production of pro-inflammatory cytokines. Studies have shown that the consumption of HT051, a combination of PL and RG, significantly downregulates adipogenic and pro-inflammatory genes in OVX rats, which leads to a reduction in adipose tissue inflammation (Figure 7).

### 5.6 Medicinal plants ameliorate liver health of post-menopausal women

Emerging evidence indicates that hepatic steatosis (fatty liver), a key metabolic consequence of obesity, is a precursor to obesity-related diseases like hyperlipidemia (Katsiki et al., 2016). There is a close relationship between total body fat and serum lipid and glucose levels, as excess adipocytes disrupt glucose and lipid homeostasis (Luna-Luna et al., 2015). FFAs from adipose tissue, released during excessive fat storage, increase serum TG and TC levels. Additionally, elevated FFAs may enhance hepatic gluconeogenesis, contributing to increased glucose production (Lam et al., 2003). Estrogen directly influences liver lipid metabolism, and its reduction is linked to an accelerated accumulation of body weight and an increase in fat mass and hepatic steatosis, marked by increased liver weight and elevated alanine aminotransferase (ALT) and aspartate aminotransferase (AST) levels (Shimizu and Ito, 2007). However, administration of estrogen-like compounds or dietary medicinal plant extracts with estrogenic properties in OVX rats has significantly reduced the serum lipid and glucose levels, which suggests that dietary phytoestrogens can prevent hyperlipidemia and hyperglycemia in post-menopausal obesity. In our review, medicinal plant extracts such as LC, GM, DC, and HT051 significantly reduced hepatic steatosis by reducing lipid size in hepatocytes, liver weight, and TC and TG levels. In addition, ALT and AST, key liver injury markers, remained within the normal range following treatment with these plant extracts in OVX rats. Compared with the Sham group, the OVX group exhibited significantly higher liver weight and serum AST and ALT levels, which were significantly lower in OVX rats fed with HT051, aqueous extracts of isoflavone-enriched soy leaves. PPARα, a transcription factor predominantly expressed in the liver, regulates lipid metabolism (Wang et al., 2020). Carnitine palmitoyltransferase 1A (CPT 1A) is crucial for fatty acid oxidation, facilitating the conversion of lipids to energy, while peroxisomal acyl-coenzyme A oxidase 1 (ACOX1) initiates the fatty acid beta-oxidation pathway, which catalyzes the desaturation of acyl-CoAs to 2 trans-enoyl-CoAs. Activated PPARα can regulate the expression of genes associated with fatty acid beta-oxidation (*ACOX1* and *CPT-1A*), thus facilitating lipid degradation (van Raalte et al., 2004). In estrogen-deficient rats, supplementation with soy, DC, and HT051 increased the expression of these genes, suggesting that these plants improve liver health by preventing the reduction in liver PPARα, CPT-1A, and ACOX1 and promoting fatty acid oxidation.

In summary, these findings collectively suggest that the aforementioned medicinal plants may be a suitable functional food source in preventing obesity-related complications during menopause due to their anti-obesity and liver-protective actions.

## 6 Clinical trial

Experimental studies have demonstrated that extracts from medicinal plants exhibit anti-obesity effects in the post-menopausal state. However, the transition from experimental to clinical application is crucial. Clinical trials play a vital role in identifying novel treatments and approaches for disease management, including early detection, diagnosis, and risk reduction strategies. The willingness of individuals to participate in such trials hinges on their understanding of the potential risks and benefits, both at a personal level and in terms of wider societal impact (Novitzke, 2008). Clinical investigations of pleiotropic natural products have demonstrated their favorable tolerability, safety, and efficacy in various medical conditions. Considering the limited range of FDA-approved obesity treatments, the exploration of new drugs, particularly those derived from medicinal plants, is essential. Medicinal plants are often considered attractive due to their potential for fewer side effects. Consequently, it is imperative to subject the aforementioned medicinal plants to rigorous clinical trials to determine their efficacy and safety. Despite the extensive exploration of these medicinal plants through animal and cellular studies, there is a notable scarcity of clinical trials.

Table 3 provides further clinical trial evidence supporting the efficacy of plant-based treatments on post-menopausal obesity. In a study by Ibrahim et al., the metabolic effects of NS were investigated in thirty menopausal women aged 45 to 60. The treatment group was orally administered 1 g of NS daily after breakfast for 2 months, whereas the control group was given a placebo. Hyperglycemia, a marker of increased obesity risk and diabetes, was significantly reduced following 8 weeks of NS administration, as evidenced by a decrease in fasting blood glucose concentration (FBGC). Additionally, since BMI is positively correlated with obesity and high BMI is associated with elevated TC (Alasmari et al., 2017).

**TABLE 3:** Clinical studies for different plants and their phytoestrogen in post-menopausal obesity.

Plant	Extract/compound	Model	Mechanisms	Ref.
*Trifolium pratense* L	isoflavone	women aged 45–60	body weight↓, TC↓, HDL↑, LDL-C↓, TG↓	Chedraui et al. (2008)
*Nigella sativa* L	isoflavone	women aged 45–60	TC↓, LDL-C↓, LpA↓, TG↓	Ibrahim et al. (2014)

TC: Total cholesterol; TG: Triglyceride; HDL: High-density lipoprotein; LDL-C: low-density lipoproteins-cholesterol; LpA: Lysophosphatidic Acid A.

NS administration results in lowered TC levels. Obesity is often characterized by increased levels of LDL-C and decreased levels of HDL-C. The treatment with black cumin capsules significantly decreased LDL-C and increased HDL-C after 8 weeks. Moreover, black cumin also notably reduced TG content, which is often elevated in overweight or obese individuals. In another study by Chedraui et al., the impact of isoflavone supplementation from TP on the lipid profile of post-menopausal women with higher BMI was examined (Chedraui et al., 2008). TP isoflavone supplementation positively influenced the lipid profile by significantly reducing TC, LDL-C, and fasting blood glucose levels. Phytoestrogen supplements have been observed to lower LDL-C, enhance liver LDL receptors, and inhibit endogenous cholesterol formation by suppressing 7α-hydroxylase activity. While isoflavone supplementation led to a reduction in BMI in women, the change did not reach statistical significance. Given the limited number of clinical studies on post-menopausal obesity, we have summarized the clinical trial data from clinicaltrials.gov in Table 4.

**TABLE 4:** Clinical study data from clinicaltrials.gov.

Study title	Conditions	Interventions	NCT number	Sponsor	Study type
Effect of Upper Limb Ergometer on the AIP in Post-Menopausal Obese Women	Post-Menopausal Obese Women	Device: Electronic Upper limb (body) ergometer	NCT04676074	Cairo University	Interventional
Effect of (Poly)Phenolic on Cardiometabolic Risk of Postmenopausal Women	Post-menopausal Women Overweight and Obesity	Combination Product: (poly)phenols rich foods	NCT05255367	Universidad de Murcia	Interventional
Obesity Prevention After Smoking Cessation in Menopause	Obesity Menopause	Behavioral: Individualized dietary-control and exercise program Behavioral: Weight-management and smoking cessation maintenance Behavioral: Smoking Cessation program	NCT00064961	National Institute on Aging (NIA)	Interventional
Effects of Sensory Motor Training on Balance and Proprioception Among Post-Menopausal Obese Women	Post Menopaused Female	Other: Sensorimotor training exercises Other: Without Sensorimotor training exercises	NCT04820738	Riphah International University	Interventional
Lung Functions in Menopausal Obese Women After COVID-19 Recovery	Lung Function Decreased Obesity Menopause	Device: Spirometry	NCT05008991	Badr University	Observational
Celecoxib Inhibition of Aromatase Expression and Inflammation in Adipose Tissue of Obese Postmenopausal Women	Obesity	Drug: Celebrex	NCT01901679	Rockefeller University	Interventional

NCT, number: The National Clinical Trial number.

Although the use of natural products as therapeutic agents for individuals with higher BMI and abnormal lipid profiles is a promising and appealing option, further research is essential. To effectively transform these natural products into medical treatments, it is suggested to integrate them with psychobehavioral interventions, pharmaceutical drugs, and mobile medical applications (Sen and Chakraborty, 2017). Additionally, capitalizing on advancements in modern technology is crucial. Developments in synthesis, fermentation, pharmacology, and pharmacodynamics, combined with the vast biological and chemical diversity and innovative evolutionary techniques or concepts, can contribute significantly. Utilizing this knowledge about medicinal plants can lead to the creation of an extensive compound library, which is invaluable for efficient drug screening (Yuan et al., 2016). Such an approach could notably advance personalized medicine and disease prevention strategies. However, it is important to acknowledge that developing modern medical research based on these medicinal plants presents various challenges and complexities that need to be addressed.

## 7 Discussion

This review highlights the potential of medicinal plants and phytoestrogens in combating obesity and metabolic dysfunction in postmenopausal women by targeting key regulatory pathways involved in adipogenesis, lipogenesis, lipolysis, and inflammation. The findings align with prior research, reinforcing the critical role of estrogen in metabolic homeostasis and the potential of plant-based estrogenic compounds to mitigate the adverse effects of estrogen deficiency.

The results demonstrate that phytoestrogen-rich medicinal plants, such as LC, GM, DC, and HT051, exhibit estrogen-like effects, reducing adipocyte hypertrophy and hyperplasia in OVX models. These effects are supported by studies that have shown that estrogen deficiency leads to increased visceral fat accumulation and dyslipidemia (Kaaja, 2008; Sirotkin and Harrath, 2014). The observed upregulation of ERα and ERβ in response to medicinal plants aligns with previous findings indicating that phytoestrogens can interact with estrogen receptors to modulate metabolic and inflammatory pathways (Wei et al., 2012; Kono et al., 2014).

In terms of lipid metabolism, our review confirms that medicinal plants can significantly regulate adipogenic and lipolytic markers, including PPARγ, aP2, SREBP-1c, and Plin. The inhibition of these transcription factors by DC and HT051 supports earlier work demonstrating the role of estrogen in suppressing adipogenesis through PPAR inhibition and mTOR activation (Mayes and Watson, 2004). Furthermore, the upregulation of CPT-1A and ACOX1 suggests enhanced fatty acid oxidation, a mechanism consistent with the known function of estrogen in promoting lipid metabolism (van Raalte et al., 2004). Notably, the reduction in hepatic steatosis observed with medicinal plants administration echoes previous studies linking estrogen deficiency with increased fat deposition and impaired lipid metabolism in the liver (Shimizu and Ito, 2007; Wang et al., 2020).

Another critical aspect of our findings is the impact of medicinal plant extracts on thermogenesis and WAT browning. The upregulation of thermogenic markers, such as UCP-1, PRDM16, and PGC-1α, following SA treatment suggests that these compounds may promote energy expenditure, a strategy previously suggested for combating postmenopausal obesity (Ikeda and Yamada, 2020). These findings extend prior knowledge by demonstrating that phytoestrogens may enhance mitochondrial activity and WAT browning, further reinforcing their potential role in metabolic regulation.

Despite the promising results presented in this review, several limitations must be acknowledged. One of the primary challenges in interpreting these findings is the variability among the included studies. Differences in experimental designs, dosages, treatment durations, and animal models may impact the comparability of results. For instance, while OVX mice are a widely accepted model for studying post-menopausal obesity, variations in surgical procedures, dietary interventions, and environmental conditions may introduce inconsistencies across studies.

Additionally, most of the reviewed studies have been conducted in animal models and *in vitro* systems, which may not fully replicate the metabolic complexities observed in human subjects. Although the results provide valuable mechanistic insights, further clinical trials are necessary to confirm the efficacy and safety of these plant extracts in post-menopausal women. Future research should focus on well-designed human studies that account for genetic, lifestyle, and environmental factors influencing obesity and metabolic disorders.

Another limitation is the lack of comprehensive dose-response studies. While the reviewed studies demonstrate the efficacy of medicinal plant extracts in modulating metabolic pathways, optimal dosages for clinical applications remain unclear. Future research should aim to establish standardized dosing regimens and evaluate potential side effects associated with prolonged phytoestrogen consumption. There is a widespread perception among consumers worldwide that medicinal plants are inherently safe because they are considered “natural” or close to nature. However, evidence indicates otherwise (Haq, 2004). Improper dosage and extended usage of such medications can lead to adverse effects. For instance, Ekaluo et al. (2011) reported that an 8-week administration of soy caused significant hormonal imbalances in rats, including reduced testosterone and Follicle-Stimulating Hormone (FSH) levels, alongside increased estradiol, LH/ICSH, and prolactin levels. Additionally, the presence of heat-labile protease inhibitors in soybeans, which require heat treatment or processing to mitigate their antinutritional effects, poses another concern. These protease inhibitors can induce pancreatic hypertrophy and hyperplasia in the short term and even lead to pancreatic nodules and carcinomas upon prolonged exposure. Furthermore, potential adverse effects linked to components like phytic acid and saponins should not be overlooked, emphasizing the need for caution and thorough evaluation when considering long-term soybean consumption (Liener, 1995).

Similarly, the consumption of TP has raised concerns regarding its safety when used over prolonged periods. Coagulation disorders have been reported, as illustrated by a case involving a 28-year-old woman who experienced severe bleeding symptoms, including gross hematuria and ecchymosis, after consuming TP and alfalfa supplements for 2 weeks. Laboratory tests indicated prolonged prothrombin time, activated partial thromboplastin time (aPTT), and an international normalized ratio exceeding seven. Notably, warfarin was detected in her blood serum despite no prior use of anticoagulants, suggesting that TP, a natural source of coumarin, can elevate international normalized ratio (INR) levels and increase bleeding risk (Karimpour-Reihan et al., 2018). Likewise, the prolonged use of basil-clove has demonstrated potential health risks, particularly when administered in high doses. Toxicological studies on Wistar albino rats revealed significant biochemical, hematological, and histopathological changes after 4 weeks of oral extract administration, emphasizing the need for caution when using this plant in herbal medicine. Additionally, subchronic toxicity assessments of hydroethanolic leaf extracts of OG in Wistar rats suggested that while acute and subchronic treatments at doses of 500 and 1,000 mg/kg appeared safe, chronic use may require monitoring of specific health parameters to avoid adverse effects (Assih et al., 2022).

Moreover, while these findings highlight the potential risks associated with the prolonged use of certain medicinal plant extracts, it is essential to consider other factors contributing to their therapeutic efficacy and safety in managing obesity. This review primarily focuses on the role of estrogen-like plant extracts in obesity prevention, but factors such as gut microbiota composition, hormonal fluctuations, and physical activity levels should also be considered. Emerging evidence suggests that gut microbiota plays a significant role in metabolic regulation and may interact with phytoestrogens to influence obesity-related outcomes (Luna-Luna et al., 2015). Investigating the interplay between phytoestrogens, gut microbiota, and metabolic health may provide a more comprehensive understanding of their therapeutic potential. The long-term anti-obesity effects of medicinal plant extracts depend on their bioactive compounds, mechanisms of action, and sustained metabolic influence. Many plant-derived compounds, such as polyphenols, alkaloids, saponins, and flavonoids, contribute to weight management through various pathways, including appetite suppression, enhanced thermogenesis, inhibition of adipogenesis, and modulation of gut microbiota, as illustrated in Figure 1 (Saad, 2023).

Sustained consumption of certain plant extracts has been shown to regulate lipid metabolism, reduce fat accumulation, and improve insulin sensitivity, leading to prolonged weight control. However, the efficacy and safety of long-term use requires rigorous clinical validation. Chronic administration may also induce adaptive metabolic responses, necessitating periodic assessment of dosage and potential side effects. Further research, including large-scale human trials, is essential to establish the long-term benefits and risks of plant-based anti-obesity interventions.

## 8 Conclusion and future perspective

The consumption of medicinal plants rich in isoflavones has been on the rise in recent decades due to their estrogenic effects. Soy products, in particular, have gained significant popularity among women for managing menopause and associated symptoms (Vincent and Fitzpatrick, 2000). *Panax ginseng* C.A.Mey., including its variants like white and red ginseng, contains saponins, primarily glycosides of triterpenoid aglycones (Ying et al., 2022). These compounds have effects comparable to estradiol and could be potential alternatives for treating post-menopausal obesity. Other medicinal plants, such as *Flemingia macrophylla* (Willd.) Merr., *Vigna unguiculata* (L.) Walp., and *Cimicifuga racemosa* (L.) Nutt., have shown promise in improving menopausal symptoms (Wong et al., 2022) and warrant thorough investigation for their potential to alleviate post-menopausal obesity.

Our study indicates that all the aforementioned plants significantly improve post-menopausal obesity both *in vivo* (Figure 6) and *in vitro* (Figure 7). Further clinical trials are needed to validate the estrogenic effects of these medicinal plants in facilitating post-menopausal weight loss. Our review acknowledges that while several plants have been studied for their potential to mitigate post-menopausal weight gain or obesity, there is a need for more well-designed randomized controlled trials. These trials should employ standardized therapies in predetermined doses over extended periods before definitive conclusions can be drawn.

## Abbreviations

HRT, Hormone replacement therapy; 17*β*-estradiol (E_2_); WAT, White adipose tissue; BAT, Brown adipose tissue; FDA, Food and Drug Administration; BMI, Body mass index; FAs, free fatty acids; GLUT4, glucose trans porter 4; UCP-1, uncoupling protein 1; AMPK, AMP-activated protein kinase; VMH, ventromedial hypothalamus; SNS, Sympathetic nervous system; OVX, Ovariectomize; PPARs, Peroxisome proliferator-activated receptors; C/EPBs, CCAAT/enhancer binding proteins; aP2, Adipocyte protein 2; LPL, lipoprotein lipase; CD68, Cluster of differentiation 68; TNF α, Tumor necrosis factor-alpha; IL-6, Interleukin 6; LDL, Low-density lipoprotein; TGs, Triglycerides; CHD, Chronic heart disease; LC, *Lycium chinense*; OG, *Ocimum gratissimum*; EU, *Eucommia ulmoides*; PL, *Pueraria lobata*; RG, *Rehmannia glutinosa*; CO, *Cornus officinalis*; DC, *Daucus carota*; ERs, ERα and ERβ; ESR1, Estrogen receptor alpha; ESR2, Estrogen receptor beta; RF, *Ribes fasciculatum*; CO + RF, Combination; MetS, Metabolic syndrome; LDL-C, Low-density lipoprotein cholesterol; HDL, High-density lipoprotein; TC, Total cholesterol; GSGE, Germinated soy germ extract; LD, Lipid droplets; SREBP-1c, Sterol-regulatory-element-binding protein 1c; FABP4, Fatty-acid-binding protein; FAS, Fatty acid synthase; Plin, *perilipin*; ATP, Adenosine triphosphate; SCD1, Stearoyl-CoA Desaturase 1; ACC, Acetyl CoA carboxylase; TAGs, Triacylglycerols; FFAs, Free FAs; ATGL, Adipose triglyceride lipase; *FSP27*, *Fat-specific protein 27*; *Plin1*, Perilipin 1; ACSL1, Acyl-CoA Synthetase Long Chain Family Member 1; ECS, Enhanced endocannabinoid system; MAGL, Monoacylglycerol lipase; PRDM16, PR domain containing 16; PGC-1α, Peroxisome proliferator-activated receptor-gamma coactivator; Tbx1, T-box transcription factor; ALT, Elevated alanine aminotransferase; AST, Aspartate aminotransferase; CPT 1A, Carnitine palmitoyltransferase 1A; ACOX1, Peroxisomal acyl-coenzyme A oxidase 1; FBGC, Fasting blood glucose concentration.
